# Efficient and explainable histopathology for cancer detection using dual-teacher distillation and integrated gradients

**DOI:** 10.3389/fmed.2026.1724419

**Published:** 2026-02-16

**Authors:** Khubab Ahmad, Saad Arif, Muhammad Hanif, Nazik Alturki, Muhammad Nabeel Asghar, Munam Ali Shah

**Affiliations:** 1 Faculty of Engineering and Technology, Multimedia University, Melaka, Malaysia; 2 Department of Mechanical Engineering, College of Engineering, King Faisal University, Al-Ahsa, Saudi Arabia; 3 Department of Informatics, School of Business, Örebro Universitet, Örebro, Sweden; 4 Department of Information Systems, College of Computer and Information Sciences, Princess Nourah bint Abdulrahman University, Riyadh, Saudi Arabia; 5 Department of Information Systems, College of Computer Science and Information Technology, King Faisal University, Al-Ahsa, Saudi Arabia; 6 Department of Computer Networks and Communication, College of Computer Science and Information Technology, King Faisal University, Al-Ahsa, Saudi Arabia

**Keywords:** deep learning, digital pathology, explainable AI, gastric cancer, histopathology, Integrated gradients, knowledge distillation, MobileNet-V2

## Abstract

Gastric cancer remains one of the most common malignancies worldwide. The timely and accurate histopathological diagnosis plays a critical role in effective treatment. Manual interpretation of histology slides is time consuming and requires considerable expertise. To address these challenges, this study introduces a two-teacher one-student (2T–1S) knowledge distillation framework for gastric cancer classification using the GasHisSDB dataset. The framework leverages DenseNet-121 and ResNet-50 as teacher networks to guide a lightweight MobileNet-V2 student. This approach provided high accuracy with significantly reduced computational cost. Experiments on multi-resolution patches (80 × 80, 120 × 120, and 160 × 160) show that the MobileNet-V2 student achieved accuracies of 95.78%, 97.46%, and 98.33%, respectively. Also, the teacher model DenseNet-121 achieved the accuracies of 96.44%, 98.75% and 98.19% and the ResNet-50 teacher reached 96.63%, 97.87% and 98.31% respectively. In addition, the student network was more than thirty times smaller and nearly twice as fast during inference. This fast light-weight model is well-suited for real-time inference on resource-constrained devices. Integrated Gradients were applied to explain the model was paying attention to actual features and focus on meaningful regions like nuclei clusters and gland boundaries. Compared with many existing techniques this framework act as balance trade-off between accuracy, speed and interpretability. This balance positions the framework as a viable tool for digital pathology workflows and further refinement could extend its utility to clinical decision support.

## 1 Introduction

Digital pathology is making modern healthcare by converting whole-slide images (WSIs) into digital form. Digitizing these WSIs enable the analysis through artificial intelligence (AI). This advancement reduces the need for traditional microscope-based examination. Also, it can improve both accuracy and consistency in diagnoses. Furthermore, the technology strengthens telepathology capabilities by allowing experts to assist patients in underserved areas (1–3). This progress provides sustainable healthcare in which AI supports pathologists and streamlines daily routines. Regulatory approval tends to progress slowly. Data security is an unresolved concern. Hospital systems are not always easy to adapt to new digital infrastructure. Finally, the limited interpretability of AI predictions continues to raise caution among clinicians (1, 4).

Deep learning has introduced progress to histopathology, especially in the analysis of digital slides. Convolutional neural networks have become a trend with well-known examples such as ResNet (5) and DenseNet (6), as well as lighter families such as MobileNetV2 (7) and EfficientNet (8). These systems have shown strong results in image classification. Large models often require powerful hardware, long processing times and substantial memory. These models have limitation in telepathology or smaller edge devices (9). These limitations have raised concerns about architectures that have high diagnostic accuracy while reducing computational power.

Knowledge distillation (KD) was introduced by Hinton et al. (10) and provides an effective solution by transferring the knowledge from a large teacher network to a smaller student model. Through this process, the student model learns not only from the hard labels but also from the soft probabilistic outputs of the teacher, leading to richer representation learning (11). Surveys of KD (11, 12) emphasize its effectiveness in compressing deep models without significant loss of accuracy. Recently, KD has been applied in medical imaging (13–15), offering lightweight yet accurate alternatives suitable for clinical practice.

In this study, mainly focus on gastric cancer detection using the Gastric Histopathology Sub-database (GasHisSDB) (16), which provides multi-resolution histopathology patches at 80 × 80, 120 × 120, and 160 × 160 pixels. Prior works on this dataset employed ensemble learning (17) and hybrid transformer approaches (18), both of which achieved high accuracy but at the expense of computational efficiency. To address this challenge a two teacher one student (2T–1S) knowledge distillation framework is introduced where DenseNet-121 and ResNet-50 act as the teachers while MobileNetV2 serves as the lighter student model. This strategy integrates the accuracy of deep networks with the efficiency of compact models with the goals of sustainable AI in digital pathology. Furthermore, interpretability is incorporated through Integrated Gradients (19), ensuring transparency in student predictions by highlighting diagnostically meaningful regions. The structure of the rest of the paper is arranged as follows: Section 2 presents the literature related to this paper, Section 3 entails the dataset and data pre-processing methodologies, Section 4 presents the utilized methodology along with the design and architecture of the proposed 2T–1S KD model, Section 5 discuss about the performance parameters that are utilized for this study and reports the obtained results. And a conclusion of this study has been given in Section 6.

## 2 Related work

The automation of histopathology analysis initially relied on classical machine learning with handcrafted features. Doyle et al. (20) extracted gray-level, Haralick, Gabor, Voronoi, and nuclear features, which were filtered with spectral clustering before SVM classification, achieving over 95% accuracy in breast cancer grading. Kather et al. (21) explored a pipeline that used handcrafted descriptors such as local binary patterns and gray level co occurrence matrices with standard classifiers. The approach reached 98.6% accuracy in a binary colorectal cancer task. The performance is impressive yet the heavy reliance on handcrafted features may restrict how well the method carries over to new datasets or cancer subtypes.

Deep learning and especially CNNs changed the field by allowing end to end feature learning. These models now show strong performance in tissue classification. (22–24). For gastric cancer the GasHisSDB dataset is widely used as a benchmark across different patch sizes (16). Studies have reported high performance using ensembles (17), hybrid networks (18) and transformers (25). These studies deliver high accuracy but often reduce efficiency in computation. To address limited dataset augmentation techniques can be used such as flipping, rotation and scaling (26–28). Transfer learning has also enabled the adaptation of pre-trained models to pathology tasks without starting from scratch with larger dataset(29, 30). Complementary studies outside histopathology provide additional insight. In addition COVID-19 detection from chest X-rays have also demonstrated how transfer learning combined with data augmentation can enhance accuracy (31).

Knowledge distillation offer a practical way to balance accuracy with computational efficiency. First introduced by Hinton et al. (10) it works by transferring knowledge from a large teacher network to a smaller student model. The student can retain much of the teacher's predictive ability while requiring less computation. Evidence suggests KD can be applied across medical imaging and related areas (11–13). In histopathology it has been used for tasks such as tissue analysis (15), segmentation (14) and survival prediction (23). The technique also appears useful beyond medicine. For instance, lightweight intrusion detection systems have leveraged KD to improve efficiency without losing accuracy (32). These studies indicate that KD can create compact models that are more feasible to deploy on platforms with limited resources in telepathology.

Interpretability is necessary for clinical environment. Gradient-based visualization methods such as Grad-CAM (33) and Integrated Gradients (19) have become standard for highlighting important tissue regions that aligning AI predictions with pathologist reasoning. In digital pathology, they have been used to emphasize nuclei, glandular structures, and tumor margins to increase accuracy in automated systems (34, 35). the use of gradcam can be observed in other medical field other than histopathology. As demonstrated in COVID-19 X-ray analysis (31) and saliency-driven ophthalmic diagnosis (36). In KD frameworks interpretability serves a dual role. Validating the student models meaningful representations and enhancing user confidence in deployment.

This study improves previous work by utilizing a multi-resolution knowledge distillation framework for gastric cancer detection. Earlier GasHisSDB studies used ensembles or transformers (17, 18). The two teacher one student (2T–1S) approach provides a light-weight student model distilled from well-performing teachers. This student can achieve accuracy that may be sufficient for clinical use while reducing computational demands. Incorporating Integrated Gradients adds interpretability and allows insight into which tissue regions influence predictions. This combination offers both reliable performance and transparency and providing suitable approach for deployment in digital pathology and telepathology environments.

## 3 Data preparation

### 3.1 Dataset description

**Figure 1:**
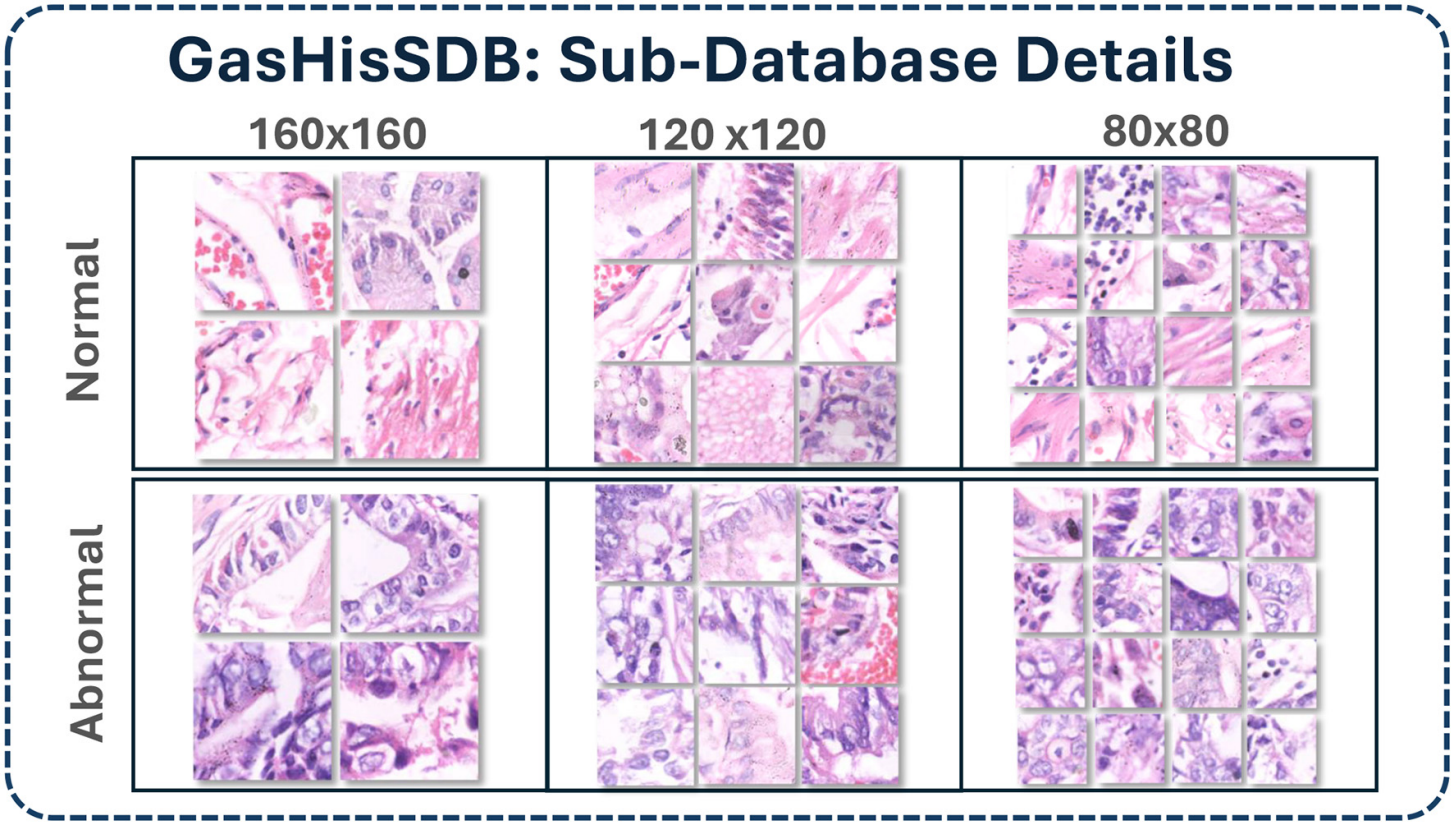
Details of GasHisSDB dataset.

The gastric histopathology sub-database GasHisSDB was used in this study. The database was created through collaboration between Longhua Hospital and Shanghai University of Traditional Chinese Medicine along with Northeastern University and the Liaoning Cancer Hospital and Institute. This collaboration ensures that clinical labels are reliable and well validated. GasHisSDB contains 600 whole slide histopathological images of gastric tissue. Each image has a resolution of 2048 × 2048 pixels. All slides were stained with hematoxylin and eosin (H&E) and captured at a magnification of × 20. For model development, each whole slide image was divided into non overlapping patches to construct the working dataset. This process produced a total of 245,196 image patches. Among them 97,076 were abnormal (cancerous) and 148,120 were normal. All patches were carefully and independently verified by expert pathologists to ensure the precision of the labeling. Furthermore, the data set was organized into three sub-databases according to patch resolution: 80 × 80 pixels, 120 × 120 pixels, and 160 × 160 pixels as Shown in Figure 1. A detailed breakdown of the patch distribution for each sub-database is presented in Table 1.

**Table 1:** Distribution of gastric tissue patches in GasHisSDB for each sub-database.

**Sub-database name**	**Cropping size**	**Abnormal**	**Normal**
Sub-database A	160 × 160 pixels	13,124	20,160
Sub-database B	120 × 120 pixels	24,801	40,460
Sub-database C	80 × 80 pixels	59,151	87,500
Total	–	97,076	148,120

This data illustrates the class distribution in each sub-database and shows skewness toward normal tissue. This imbalance reflects real-world biopsy data. The dataset was prepared following strict protocols to maintain diagnostic reliability. Patches of normal tissue were directly cropped from WSIs labeled as non-cancerous and abnormal patches were extracted from cancerous regions annotated by pathologist. Any patch containing less than 50% malignant tissue was excluded to prevent mislabeling. All patches were randomly rotated and shuffled to reduce correlations between neighboring patches of same whole slide image. This strategy significantly improves the model's ability to generalize.

The GasHisSDB dataset is commonly used in gastric cancer detection studies and provides a reliable benchmark for evaluating deep learning approaches. Its structure features multi-resolution patches that highlight the relationship between local details and broader context. This provide the balance comparison between fine-grained features and overall tissue patterns in histopathology classification tasks.

### 3.2 Empty patch removal process

**Figure 2:**
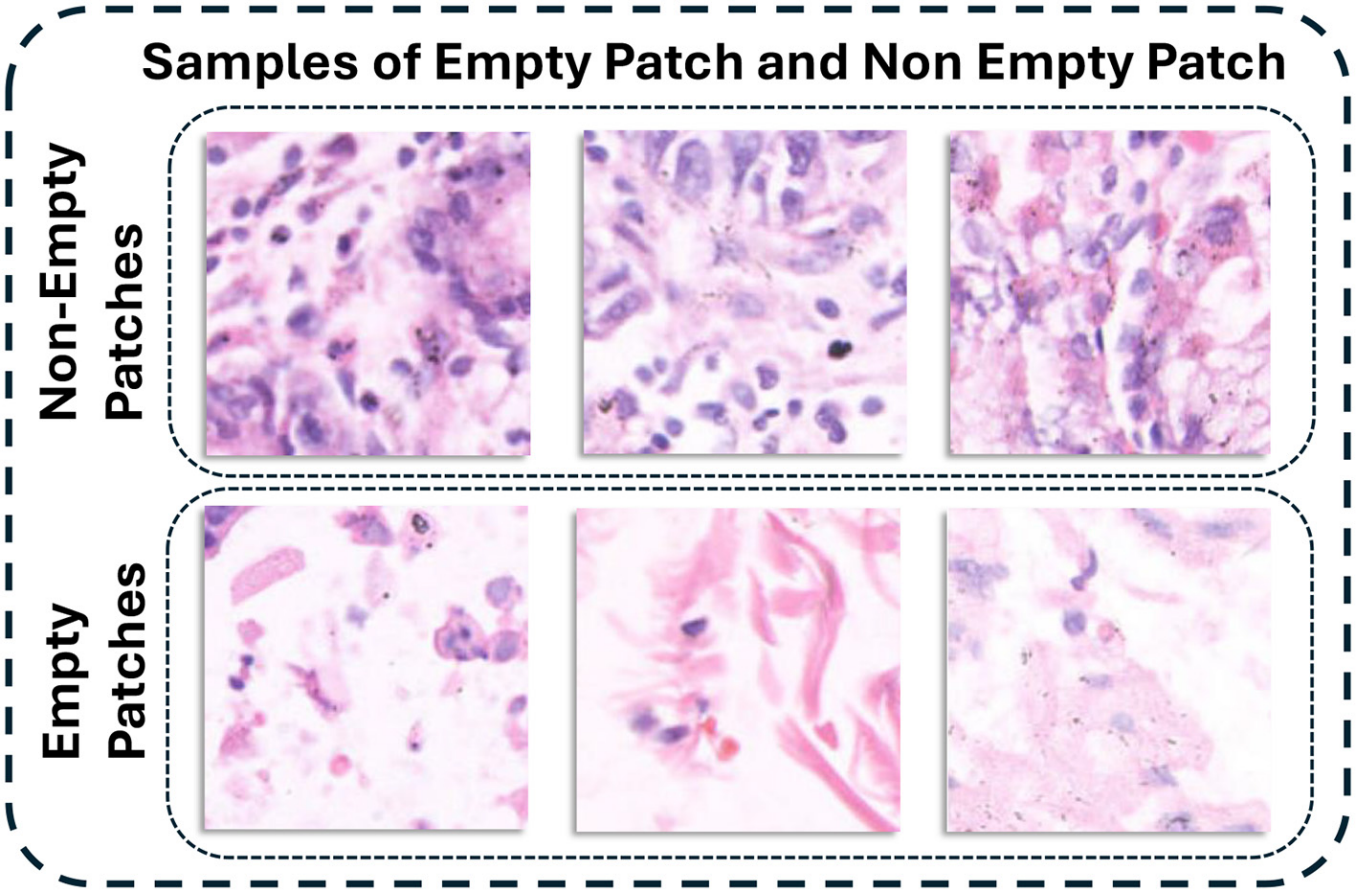
Comparison of empty and non-empty patches.

In this dataset many histopathology patches are still be non-diagnostic due to background whitespace or include tissue folds. There are also staining regions or areas that offer little useful information for analysis. These patches can increase computational power in training process and also reduce model accuracy. Therefore these patches were cleaned for better diagnostic. The cleaning pipeline used a filter based on pixel intensity. For each patch, the proportion of pixels with an intensity value greater than 230 (out of 255) across all three RGB channels was calculated. This threshold was selected because high intensities consistently indicated background rather than cellular structure. Patches in which more than 50% of pixels exceeded this threshold were treated as non-informative and removed from the dataset. The sample patches with high white background and retained diagnostic patches are illustrated in Figure 2.

This filtering step affected the classes differently. Normal patches were removed at a higher rate due to their large homogeneous areas. Approximately 31–33% of normal patches were discarded across the three resolutions and 7–11% of cancerous patches were removed. Abnormal slides exhibit greater tissue density and nuclear concentration, which naturally results in fewer empty or unusable images.

**Figure 3:**
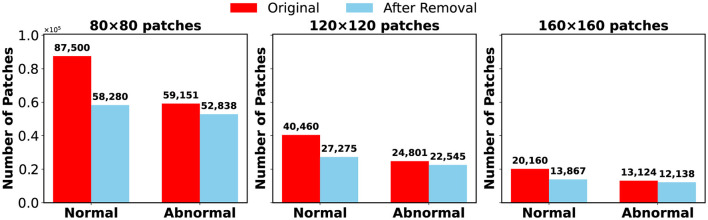
Number of patches before and after removal of each resolution.

The number of patches remaining after the cleaning step is summarized in Figure 3. This figure presents the data for the 80 × 80, 120 × 120, and 160 × 160 resolutions using bar plots. The analysis demonstrates that cleaning significantly reduced dataset size but improved overall quality by ensuring that the retained images contained meaningful histological features.

### 3.3 Data augmentation

Medical imaging datasets in histopathology often face class imbalance and limited diversity because patches are taken from a fixed set of WSIs. Despite the large number of patches in GasHisSDB many come from spatially adjacent tissue regions. This can create redundancy and reduce the model's ability to generalize. To address this challenge a data augmentation pipeline was applied to artificially increase dataset diversity, reduce overfitting and improve model robustness. The augmentation strategy was specifically designed to create realistic variations encountered in histopathological imaging while preserving the important diagnostic content.

In this study, several transformations techniques were applied to the training patches. First random rotation of 90° was used. This approach improves model performance while preserving key diagnostic details. Also, horizontal and vertical flips were included to reflect potential slide orientations. Also applied random horizontal and vertical shifts of up to 10% to mimic variations in cropping during WSI patch extraction. Finally, random zooms within a range of ±20% were applied to simulate magnification variability and enhance the scale invariance of the trained models. After augmentation all patches were normalized to the [0, 1] intensity range to ensure consistency across batches. The effect of augmentation on dataset size is summarized in Table 2. This table represent the number of patches before and after augmentation across all three resolutions (80 × 80, 120 × 120, and 160 × 160).

**Table 2:** Number of image patches in the training set before and after augmentation.

**Patch size**	**Original samples**	**After augmentation**
80 × 80	66,671	133,342
120 × 120	29,892	59,784
160 × 160	15,603	31,206

Augmentation not only increased the size of the training datasets but also ensured that the models learn on different features and spatial variations. This step was important for the student model in the KD framework because smaller models are more tend to overfitting. By presenting augmented variations the student learn more generalizable features while maintaining efficiency.

### 3.4 Dataset splitting

Following patch cleaning and augmentation, the datasets were split into training, validation and testing subsets to enable model development and unbiased evaluation. A stratified sampling strategy was used to maintain class balance across all subsets. To ensure that both normal and cancerous classes were equaly represented. TThis step was important because the raw GasHisSDB dataset showed a clear class imbalance with normal tissue patches outnumbering abnormal ones. To ensure reproducibility the random seed used for split was fixed. The resulting subsets for each patch resolution are summarized in Table 3. For each resolution, approximately 60% of samples were allocated to training (with augmentation applied), 20% to validation, and 20% to testing.

**Table 3:** Dataset partitioning across different patch resolutions (60/20/20 split with training augmentation).

**Patch size**	**Augmented training set**	**Validation set**	**Testing set**
80 × 80	133,342	22,224	22,223
120 × 120	59,784	9,964	9,964
160 × 160	31,206	5,201	5,201

This split offered three clear advantages. The training set was used to adjust the weights of both teacher and student models and augmentation was applied only to this set to increase diversity and improve generalization. The validation set tracked learning progress and guided hyperparameter tuning with early stopping based on validation accuracy and loss to prevent overfitting. The testing set remained completely unseen during training and validation so performance metrics reflected true generalization rather than memorization. The relatively large test sets at each resolution guaranteed robust and statistically meaningful evaluation. Moreover, by maintaining stratified distributions across subsets, the experimental design reduced the risk of bias and produced fair comparisons between teacher and student models.

## 4 Methodology and experiments

### 4.1 Overview of the framework

**Figure 4:**
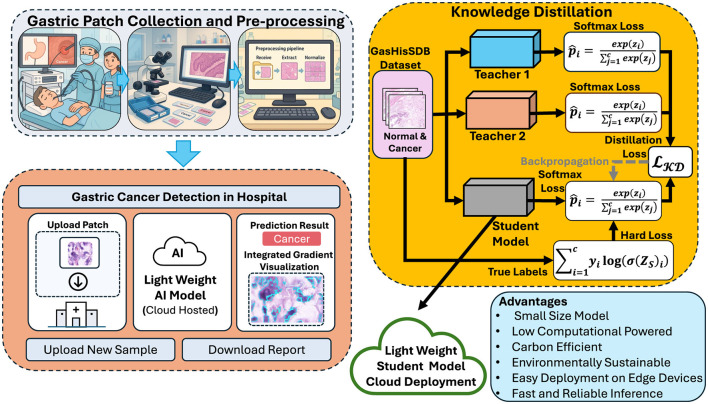
Overview of the proposed framework for gastric cancer detection. Created with Inkscape.

The proposed framework is based on the KD and follows the principles outlined in Digital Pathology and Telepathology. It is designed to transfer diagnostic skills from high-capacity teacher networks to a lightweight student model for efficient gastric cancer detection. The complete workflow is illustrated in Figure 4, which highlights the sequential steps of teacher training, student distillation and model deployment.

In the first stage teacher models are trained independently on the cleaned and augmented GasHisSDB datasets. These teachers are large and high-performing CNNs capable of capturing rich hierarchical features from histopathology images. Due to their depth and complexity teacher models achieve high accuracy but are computationally expensive, limiting their suitability for real-time clinical applications. In the second stage a compact student model is trained using knowledge of the teachers. It does not rely on the ground-truth labels the student leverages both hard labels representing the categorical ground-truth annotations (normal vs. abnormal) and soft labels which are derived from the probabilistic outputs of the teacher models by applying temperature scaling. By combining these two sources of supervision the student learns not only the correct class assignments but also the inter-class relationships captured by the teachers. This process of knowledge transfer enables the student model to reach performance levels similar to the teacher. Using fewer parameters and requiring less computation.

The final stage of the framework focuses on deployment and inference. The student model has been distilled with teachers knowledge and achieve accuracy close to the teachers. It also remains computationally lightweight. This compact student model can be used for different clinical environments such as cloud platforms for large-scale analysis or edge devices in hospital laboratories. The framework also integrates explainability methods such as integrated gradients. These methods highlight tissue regions that most influence the model's decisions. These interpretability can increase clinical decision and support validation by pathologists. Overall the knowledge distillation framework provide balance accuracy efficiency and interpretability. The training of high-accuracy teachers and transferring their knowledge to a smaller student is best compact solution for automated gastric cancer detection that is sustainable in real-world settings.

### 4.2 Knowledge distillation objective

**Figure 5:**
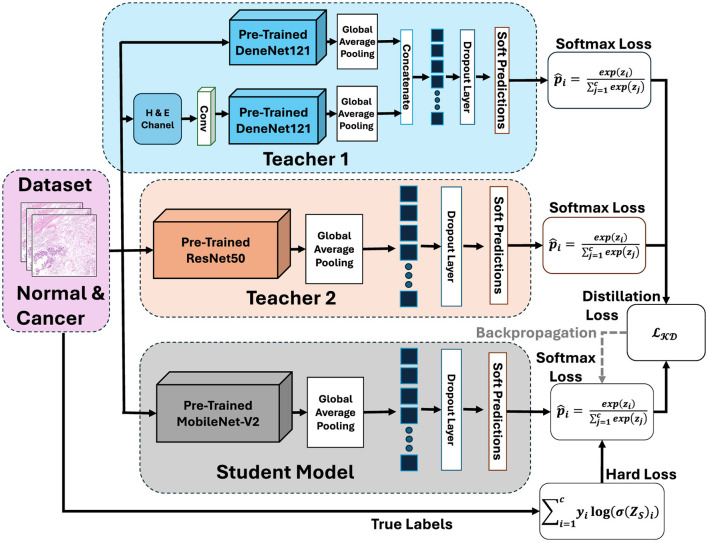
Proposed 2T–1S knowledge distillation.

The main contribution of this study is the use of a two teacher one student (2T–1S) knowledge distillation framework. Rather than relying on a single teacher the student receives knowledge from two dense and deeper networks. Teacher 1 (T1) is a densely architecture and Teacher 2 (T2) is a residual architecture. This allows the student to utilized unique strengths of both teachers. It produce a model that is more generalizable and effective for gastric cancer detection. Figure 5 illustrates the 2T–1S KD process. The diagram shows how the student receives dual supervision, hard labels from the ground-truth annotations, and soft labels from both teachers. The student's predictions are optimized by minimizing a composite loss function that integrates these signals.

#### 4.2.1 Hard label supervision

Ground-truth labels are incorporated using categorical cross-entropy loss:


$$\begin{array}{c} L_{\text{hard}}=-\overset{C}{\underset{i=1}{\sum }}y_{i}\log(S_{i}\left(x\right)), \end{array}$$ (1)

where *y*_*i*_ is the true label for class *i*, *S*_*i*_(*x*) is the student's predicted probability, and *C* = 2 (normal vs. cancer).

#### 4.2.2 Soft label supervision from two teachers

Each teacher generates softened probability distributions using temperature scaling:


$$\begin{array}{c} \sigma_{T}\left(z_{i}\right)=\frac{\exp\left(z_{i}/T\right)}{\sum_{j=1}^{C}\exp\left(z_{j}/T\right)}, \end{array}$$ (2)

where *z*_*i*_ is the logit for class *i*, and *T* = 5 is the softmax temperature applied to both teacher and student outputs.

The similarity between teacher and student distributions is captured using Kullback–Leibler (KL) divergence. To ensure stable and balanced supervision from both teacher models, their output logits were fused using equal-weight averaging. This strategy is effective for heterogeneous networks such as DenseNet-121 and ResNet-50, as it avoids domination by any single architecture and provides complementary guidance during student training. Since two teachers are employed, the distillation loss is computed separately for each teacher- student pair and then equal-weight averaged:


$$\begin{array}{c} L_{\text{KD}}=\frac{1}{2}(L_{\text{KD}}\left(T_{1},S\right)+L_{\text{KD}}\left(T_{2},S\right)), \end{array}$$ (3)

with


$$\begin{array}{c} L_{\text{KD}}\left(T,S\right)=\overset{C}{\underset{i=1}{\sum }}\sigma_{T}\left(z_{T,i}\right)\log(\frac{\sigma_{T}\left(z_{T,i}\right)}{\sigma_{T}\left(z_{S,i}\right)}). \end{array}$$ (4)

#### 4.2.3 Composite loss function

The final distillation loss combines ground-truth supervision with the averaged teacher guidance:


$$\begin{array}{c} L_{\text{total}}=\alpha \cdot L_{\text{hard}}\left(y,S\left(x\right)\right)+\left(1-\alpha \right)\cdot T^{2}\cdot L_{\text{KD}}, \end{array}$$ (5)

where α = 0.5 balances direct supervision and teacher knowledge transfer. The factor *T*^2^ used for gradient scaling at higher temperatures.

By integrating knowledge from two teachers, the student achieved higher accuracy than a single teacher setup. The 2T–1S KD strategy not only preserves classification accuracy but also enhances robustness and generalization. The distilled student is well-suited for deployment in clinical environments with limited computational resources.

### 4.3 Teacher models

The framework uses two teacher models to guide the student network. Both teachers rely on transfer learning. Their convolutional backbones are first trained on ImageNet and then fine-tuned on the GasHisSDB dataset to handle gastric histopathology images. Using this general visual features such as edges, textures and color patterns learned from natural images can be applied to the more specialized medical imaging domain.

#### 4.3.1 Teacher 1: two-branch DenseNet-121

**Figure 6:**
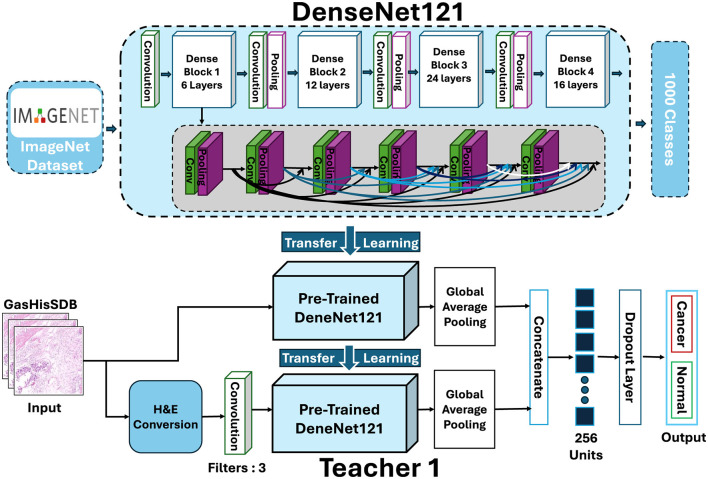
Architecture of Teacher 1 (T1): a two-branch DenseNet-121.

The first teacher (T1) is a customized two-branch DenseNet-121 model. This model designed to enhance feature diversity by processing the input images through two distinct representation streams. The architecture of Teacher 1 is shown in Figure 6, where the two-branch design integrates both RGB and H&E-based representations to improve feature learning.

Branch 1 (RGB Input) The original histopathology patches in RGB format go through the DenseNet backbone. DenseNet uses dense connections to reuse features and keep gradients stable. It can capture fine details as well as larger tissue structures. Branch 2 (H&E Conversion Stream) The same input patch is coverted into its hematoxylin and eosin (H&E) stain representation. This highlights nuclei and cytoplasmic structures that are important for diagnosis. Since DenseNet requires three-channel inputs, a 2D convolutional layer is used to turn the H&E map into a compatible format. This branch allows the model to use stain-specific information alongside the RGB features from Branch 1. The outputs of both branches are concatenated to create a comprehensive feature representation. This fusion allows T1 to simultaneously capture natural image cues from RGB patches and domain-specific histopathological cues from H&E stain maps.

After fusion, T1 has a task-specific classification head. Global average pooling condenses the high-dimensional feature maps. A dense layer with 256 units and ReLU activation transforms the features. Dropout with *p* = 0.5 helps prevent overfitting. The final softmax layer produces probabilities for the two classes, normal and cancerous. This dual-branch setup is effective because it combines raw tissue morphology with stain-enhanced features into one unified representation.

#### 4.3.2 Teacher 2: ResNet-50

**Figure 7:**
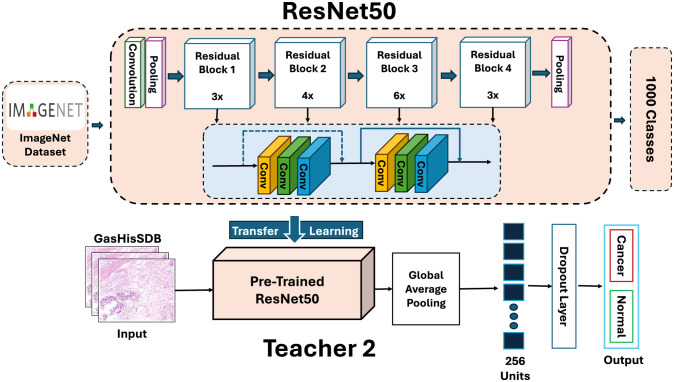
Architecture of Teacher 2 (T2): a ResNet-50.

The second teacher (T2) uses a ResNet-50 backbone that was first trained on ImageNet and then fine-tuned on GasHisSDB. Figure 7 shows the ResNet-50 architecture. Its residual connections can do hierarchical feature extraction and support more stable optimization.

T2 processes input patches through a series of residual bottleneck blocks. These blocks gradually turn low-level features into higher-level semantic representations. Like T1, T2 has a custom classification head. Global average pooling condenses the feature maps and a dense layer with 256 hidden units transforms the features. Dropout is used to prevent overfitting. The final layer is a softmax classifier for the binary prediction.

#### 4.3.3 Complementarity of teachers

Using both a two-branch DenseNet-121 (T1) and a ResNet-50 (T2) gives complementary benefits. T1 captures features from both RGB input and H&E stain maps. T2 uses residual learning to extract hierarchical features and maintain stable optimization. Together the two teachers provide the student with diverse guidance during knowledge distillation.

### 4.4 Student model

The student model (S1) is a lightweight and efficient alternative to the larger teacher networks. It aims to maintain diagnostic accuracy through knowledge distillation. To do this the model uses a MobileNet-V2 backbone. This design seems well suited for real-time inference in settings with limited resources such as clinical laboratories or edge devices.

#### 4.4.1 Backbone architecture

**Figure 8:**
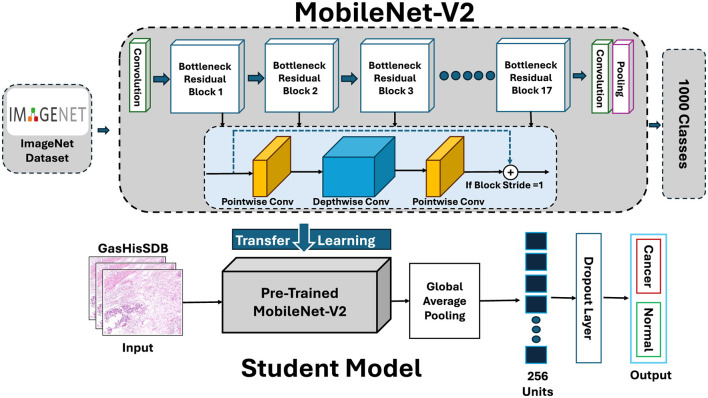
Architecture of the student model (S1) based on MobileNet-V2 backbone.

MobileNet-V2 introduces two design features that make it much more efficient than conventional CNNs. The first is Inverted Residual Blocks. Instead of expanding feature maps gradually the network projects them into a higher-dimensional space applies depthwise convolution and then compresses them back into a compact form. This improves accuracy while keeping computation low. The second feature is Depthwise Separable Convolutions. Standard convolutions are split into depthwise and pointwise (1 × 1) operations which reduces the number of trainable parameters and floating-point operations without a big drop in performance. The backbone has 17 bottleneck residual blocks that extract features from low to high levels. Identity shortcuts are added when dimensions match which helps keep gradient flow stable even in this compact design. Figure 8 shows the student model highlighting the MobileNet-V2 backbone and the lightweight classification head.

#### 4.4.2 Transfer learning and custom classification head

Like the teachers, the student backbone was initialized with ImageNet pre-trained weights. Unlike the teachers fine-tuning is used to avoid overfitting and keep the model efficient. The MobileNet-V2 backbone was adapt for binary gastric cancer classification and task-specific head is added. Global average pooling compresses the spatial feature maps into compact descriptors. A dense layer with 256 hidden units and ReLU activation transforms the features. Dropout with a rate of 0.5 helps prevent overfitting. The final softmax layer produces class probabilities for normal and cancerous patches.

#### 4.4.3 Efficiency and deployment

The distilled student model has far fewer parameters and a smaller memory footprint than its teachers. This results in quicker predictions with lower energy use. The model is suitable for edge devices such as hospital workstations or mobile diagnostic tools and for cloud platforms. Knowledge distillation allows the student to reach accuracy close to the larger teacher models without higher computational power. The student acts as a condensed version of the teacher ensemble combining efficiency with reliable diagnostic performance. Student model can be deploy as practical tool for clinical gastric cancer detection.

## 5 Experimentation and results

The experimental evaluation of the 2T–1S knowledge distillation framework is organized by patch resolution at 80 × 80, 120 × 120, and 160 × 160. The evaluation then covers model efficiency, comparison with previous studies and an assessment of interpretability.

### 5.1 Evaluation metrics

To quantitatively assess classification performance, the following metrics were employed:


$$\begin{array}{c} Accuracy=\frac{TP+TN}{TP+TN+FP+FN} \end{array}$$ (6)

$$\begin{array}{c} Precision=\frac{TP}{TP+FP} \end{array}$$ (7)

$$\begin{array}{c} Recall=\frac{TP}{TP+FN} \end{array}$$ (8)

$$\begin{array}{c} F1=2\cdot \frac{Precision\times Recall}{Precision+Recall} \end{array}$$ (9)

$$\begin{array}{c} Specificity=\frac{TN}{TN+FP} \end{array}$$ (10)

$$\begin{array}{c} NPV=\frac{TN}{TN+FN} \end{array}$$ (11)

where *TP*, *TN*, *FP*, and *FN* denote true positives, true negatives, false positives, and false negatives, respectively.

### 5.2 Results on 80 × 80 patches

#### 5.2.1 Classification performance

Table 4 shows the classification metrics for T1, T2, and S1 on 80 × 80 patches. Both teacher networks performed well, with T2 slightly ahead of T1 in recall for the abnormal class at 97.12% compared to 96.48%. This indicates that ResNet-50 is more sensitive to cancerous regions at this lower resolution. The student model has fewer parameters and delivered competitive results. It maintained a precision of 96.67% for normal patches and a recall of 96.39% for abnormal patches. Overall accuracy at 95.78% was a bit lower than the teachers, but that knowledge distillation preserved the key discriminative ability.

**Table 4:** Classification report for 80 × 80 patches (class-wise and overall).

**Model**	**Class**	**Precision (%)**	**Recall (%)**	**F1-score (%)**	**Specificity (%)**	**NPV (%)**	**AUC**
T1 (DenseNet-121)	Abnormal	96.05	96.48	96.27	96.40	96.79	0.99
	Normal	96.80	96.41	96.60	96.48	96.05	
	Overall accuracy	96.44					
T2 (ResNet-50)	Abnormal	95.85	97.12	96.48	96.19	97.36	0.99
	Normal	97.36	96.19	96.77	97.12	95.85	
	Overall accuracy	96.63					
S1 (MobileNet-V2)	Abnormal	94.82	96.39	95.60	95.23	96.67	0.98
	Normal	96.67	95.23	95.95	96.39	94.82	
	Overall accuracy	95.78					

#### 5.2.2 Confusion matrices

**Figure 9:**
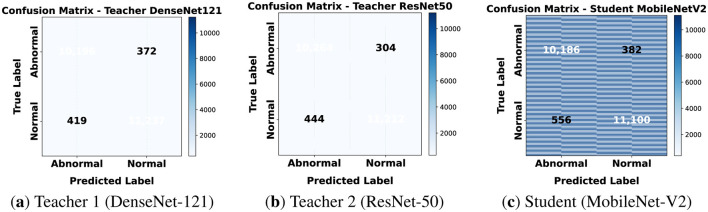
Confusion matrices for classification on 80 × 80. **(a)** Teacher 1 (DenseNet-121). **(b)** Teacher 2 (ResNet-50). **(c)** Student (MobileNet-V2).

The confusion matrices for 80 × 80 patches are shown in Figure 9. Both teachers show a balanced prediction across normal and abnormal classes with only a few misclassifications. The student has slightly more false positives and labels some normal tissue as abnormal but overall errors remain low. This suggests that knowledge distillation allowed the student to reach performance close to the teachers while using far less computation. At this resolution models mainly capture local structures such as nuclei and cytoplasmic differences. Both teachers achieved high recall confirming sensitivity to cancerous regions. The student was slightly lower in precision but still generalized reliably making it suitable for fast clinical inference on small patches.

### 5.3 Results on 120 × 120 Patches

#### 5.3.1 Classification Performance

Table 5 summarizes classification performance on 120 × 120 patches. Both teachers improved compared to the 80 × 80 case especially in precision. T1 achieved 98.75% accuracy while T2 reached 97.87%. The student model also gained from the larger context and achieved 97.11% accuracy close to its teachers. Notably S1 reached 98.26% recall on normal patches showing its robustness after distillation.

**Table 5:** Classification report for 120 × 120 patches (class-wise and overall).

**Model**	**Class**	**Precision (%)**	**Recall (%)**	**F1-score (%)**	**Specificity (%)**	**NPV (%)**	**AUC**
T1 (DenseNet-121)	Abnormal	98.47	98.76	98.62	98.74	98.97	0.99
	Normal	98.97	98.74	98.85	98.76	98.47	
	Overall accuracy	98.75					
T2 (ResNet-50)	Abnormal	98.34	96.94	97.63	98.64	97.50	0.99
	Normal	97.50	98.64	98.07	96.94	98.34	
	Overall accuracy	97.87					
S1 (MobileNet-V2)	Abnormal	97.85	95.72	96.77	98.26	96.52	0.99
	Normal	96.52	98.26	97.38	95.72	97.85	
	Overall accuracy	97.11					

#### 5.3.2 Confusion matrices

**Figure 10:**
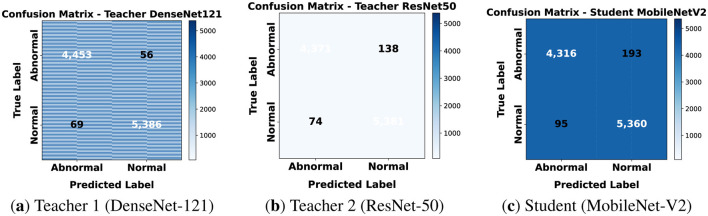
Confusion matrices for classification on 120 × 120. **(a)** Teacher 1 (DenseNet-121). **(b)** Teacher 2 (ResNet-50). **(c)** Student (MobileNet-V2).

Confusion matrices in Figure 10 confirm this trend. Both teachers make fewer mistakes compared to 80 × 80. The student occasionally mislabels abnormal patches as normal but still performs close to the teachers. This demonstrates that knowledge transfer is effective.

At this resolution both local cell-level and broader contextual features are captured. The teachers performed very well in precision and recall. The student also showed strong generalization and robustness. This confirms that larger patch sizes provide richer information benefiting both teacher and student models.

### 5.4 Results on 160 × 160 patches

#### 5.4.1 Classification performance

Table 6 reports performance on 160 × 160 patches. This resolution perform the highest for all models. T2 achieved 98.31% accuracy while S1 reached 98.33%, effectively matching its teachers. The student also kept a good balance between abnormal and normal predictions. It can generalize across different patch sizes.

**Table 6:** Classification report for 160 × 160 patches (class-wise and overall).

**Model**	**Class**	**Precision (%)**	**Recall (%)**	**F1-score (%)**	**Specificity (%)**	**NPV (%)**	**AUC**
T1 (DenseNet-121)	Abnormal	98.54	97.57	98.05	98.74	97.89	0.99
	Normal	97.89	98.74	98.31	97.57	98.54	
	Overall accuracy	98.19					
T2 (ResNet-50)	Abnormal	97.56	98.85	98.20	97.84	98.98	0.99
	Normal	98.98	97.84	98.40	98.85	97.56	
	Overall accuracy	98.31					
S1 (MobileNet-V2)	Abnormal	98.03	98.39	98.21	98.27	98.59	0.99
	Normal	98.59	98.27	98.43	98.39	98.03	
	Overall accuracy	98.33					

#### 5.4.2 Confusion matrices

**Figure 11:**
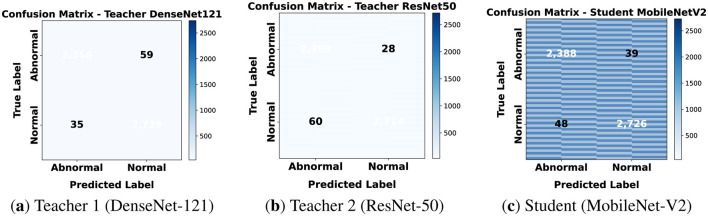
Confusion matrices for classification on 160 × 160. **(a)** Teacher 1 (DenseNet-121). **(b)** Teacher 2 (ResNet-50). **(c)** Student (MobileNet-V2).

Confusion matrices in Figure 11 highlight the strong performance of all three models at this resolution. The confusion matrices reveal very few errors in distinguishing normal from abnormal patches. The student's results closely match its teachers to confirm successful distillation.

At 160 × 160, the larger receptive field allows models to leverage both local detail and contextual tissue architecture. This improved diagnostic accuracy for all models. The student's accuracy of 98.33% demonstrates that it can serve as an efficient yet reliable replacement for heavier teacher networks.

### 5.5 Benefit of dual-teacher strategy

The effectiveness of the 2T–1S distillation framework was tested by comparing it with two single-teacher setups where the student learned from either DenseNet-121 or ResNet-50 alone. Table 7 shows that the dual-teacher configuration consistently achieves higher accuracy across all patch resolutions. Improvements ranged from about 0.8% to 1.4% compared to the strongest single-teacher baseline. This suggests that guidance from two different teachers provides a better and more transferable knowledge signal than relying on just one.

**Table 7:** Comparison of single-teacher vs. dual-teacher knowledge distillation for the student model.

**Patch resolution**	**Student (DenseNet-121 only)**	**Student (ResNet-50 only)**	**Student (2T–1S approach)**	**Improvement**
80 × 80	94.12%	94.55%	**95.78%**	+1.23%
120 × 120	95.80%	96.15%	**97.11%**	+0.96%
160 × 160	96.45%	96.95%	**98.33%**	+1.38%

Bold values indicate the highest improvement.

### 5.6 Sensitivity analysis of distillation hyperparameters

A sensitivity analysis was carried out to find the best values for the distillation temperature (*T*) and the weighting factor (α) across all patch resolutions. As shown in Table 8, the configuration *T* = 5 and α = 0.5 consistently produced the highest student accuracy, yielding 95.78% at 80 × 80, 97.11% at 120 × 120, and 98.33% at 160 × 160. Lower temperatures like *T* = 2 produced very sharp teacher outputs. This limited the transfer of soft-label information and reduced performance by about 0.4%. A weighting factor of α = 0.5 gave a balanced contribution between ground-truth labels and teacher predictions.

**Table 8:** Sensitivity analysis of distillation temperature (*T*) and weighting factor (α) on student accuracy across patch resolutions.

**Hyperparameter**	**value**	**Accuracy (80 × 80)**	**Accuracy (120 × 120)**	**Accuracy (160 × 160)**
Temperature (*T*)	2	95.38%	96.71%	97.93%
	5 (Selected)	**95.78%**	**97.11%**	**98.33%**
	10	95.58%	96.91%	98.13%
Weighting Factor (α)	0.3	95.48%	96.81%	98.03%
	0.5 (Selected)	**95.78%**	**97.11%**	**98.33%**
	0.7	95.63%	96.96%	98.18%

Bold values indicates the selected parameters.

Using *T* = 5 gave the best results across every resolution. The results show the best temperature depends on the size difference between teacher and student models instead of the spatial dimension of the input data. Regarding the weighting factor, α = 0.5 worked best by balancing teacher guidance to regularize the student while keeping strict focus on the actual ground truth labels.

### 5.7 Teacher confidence analysis

**Figure 12:**
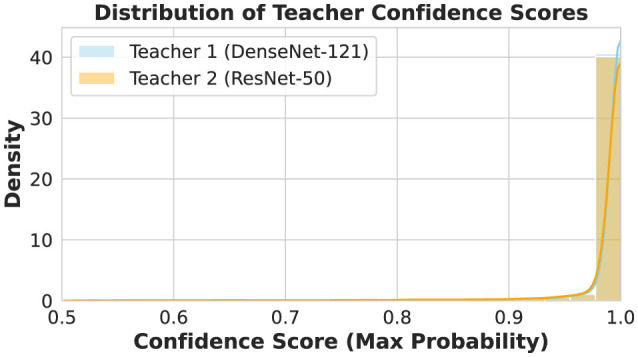
Representative distribution of confidence scores on the test set for Teacher 1 (DenseNet–121) and Teacher 2 (ResNet–50) at 160 × 160 resolution.

The study validated the fusion strategy by examining maximum softmax probabilities from both teachers. These confidence scores served as the key metric on the validation set. Figure 12 illustrates the representative confidence distributions observed for the 160 × 160 best resolution case. Both Teacher 1 (DenseNet-121) and Teacher 2 (ResNet-50) exhibited highly consistent behavior, with distributions sharply concentrated near 1.0. This indicates that both architectures achieved high confidence in their predictions. Consequently, an equal fusion weighting strategy (*w*_1_ = *w*_2_ = 0.5) was adopted to balance the supervision signals from both teachers. This ensure that student model benefits equally from the distinct feature representations of each architecture without biasing toward a specific teacher.

### 5.8 Representation similarity analysis

Representational similarity between the teacher networks and the student was measured using linear Centered Kernel Alignment (CKA), a common method for comparing feature spaces across networks with different dimensions. CKA gives a scale-invariant similarity score and works well for assessing knowledge transfer in distillation. As shown in Table 9, the results indicate that the MobileNet-V2 student captured the main diagnostic features learned by both teachers. Similarity scores for DenseNet-121 and the student ranged from 0.68–0.78, while ResNet-50 and the student showed values between 0.64–0.75. Similarity values above 0.60 denote strong alignment, suggesting that the distilled student inherits relevant discriminative structure from both teacher models. The moderate similarity observed between the two teachers themselves, DenseNet-121 and ResNet-50 (0.55–0.67), indicates that the teacher networks learn partially complementary feature spaces. This supports the use of a dual-teacher distillation strategy, as it provides a richer supervisory signal than either teacher alone.

**Table 9:** Estimated linear CKA representational similarity between teacher and student models.

**Model pair**	**CKA range**	**Interpretation**
DenseNet-121 ↔ Student	0.68–0.78	Strong alignment
ResNet-50 ↔ Student	0.64–0.75	Strong alignment
DenseNet-121 ↔ ResNet-50	0.55–0.67	Complementary feature spaces

### 5.9 Impact of empty patch removal

The effect of removing background-dominant patches was quantified by training the student model with and without this preprocessing step. Since the GasHisSDB dataset contains a substantial number of patches with minimal or no tissue content, retaining such samples introduces noise into the learning process. As shown in Table 10, excluding empty patches improved accuracy by approximately 1.3% to 2.1% across the evaluated resolutions. Training on raw, unfiltered patches often led the model toward suboptimal convergence, as empty regions labeled according to slide-level metadata provided no morphological cues for discrimination. Removing such patches ensured that the network was exposed only to informative tissue structures, thereby stabilizing optimization and improving generalization.

**Table 10:** Impact of empty patch removal on student model accuracy.

**Resolution**	**Accuracy (without removal)**	**Accuracy (with removal)**	**Improvement**
80 × 80	94.31%	**95.78%**	+1.47%
120 × 120	95.82%	**97.11%**	+1.29%
160 × 160	96.25%	**98.33%**	+2.08%

Bold values indicates accuracy after empty patch removal.

### 5.10 Model complexity and efficiency

**Figure 13:**
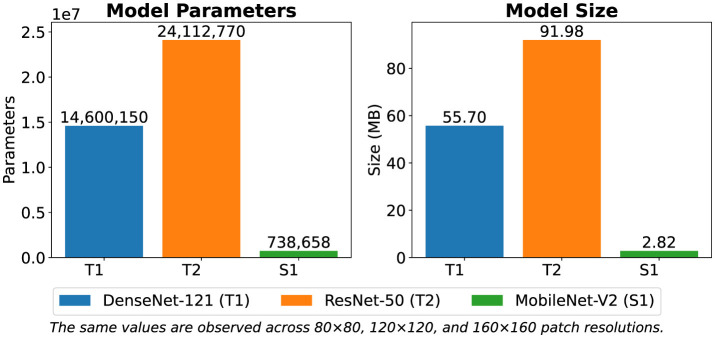
Comparison of model parameters and model size across patch resolutions (80 × 80, 120 × 120, 160 × 160).

**Figure 14:**
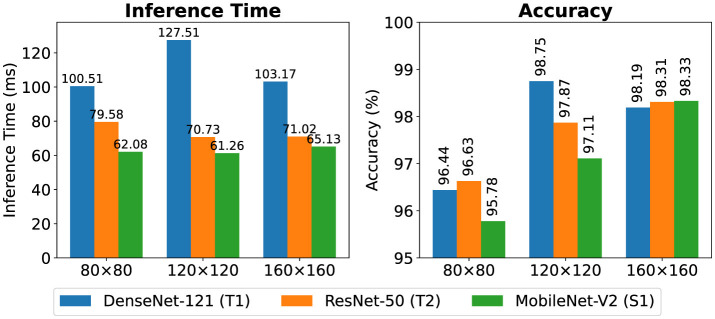
Comparison of inference time and accuracy across patch resolutions (80 × 80, 120 × 120, 160 × 160).

To evaluate computational efficiency Table 11 shows comparison of complexity metrics across patch sizes. T1 and T2 had 14.6M and 24.1M parameters respectively with memory footprints of 55.7 MB and 91.9 MB. Also, the student had fewer than 1M parameters (2.82 MB) as shown in Figure 13. This make it lightweight and suitable for resource-limited environments. In addition to reduction in size, S1's accuracy remained close to or matched its teachers across resolutions as shown in Figure 14. This confirms that the knowledge distillation approach provides a practical balance between performance and efficiency.

**Table 11:** Complexity comparison of T1, T2, and S1 across different patch resolutions.

**Patch size**	**Model**	**FLOPs (GFLOPs)**	**Total params**	**Model size (MB)**	**Inference (ms)**	**Accuracy (%)**
80 × 80	T1 (DenseNet-121)	0.95	14,600,150	55.70	100.51	96.44
	T2 (ResNet-50)	1.32	24,112,770	91.98	79.58	96.63
	S1 (MobileNet-V2)	0.12	738,658	2.82	62.08	95.78
120 × 120	T1 (DenseNet-121)	2.15	14,600,150	55.70	127.51	98.75
	T2 (ResNet-50)	2.99	24,112,770	91.98	70.73	97.87
	S1 (MobileNet-V2)	0.27	738,658	2.82	61.26	97.11
160 × 160	T1 (DenseNet-121)	3.82	14,600,150	55.70	103.17	98.19
	T2 (ResNet-50)	5.31	24,112,770	91.98	71.02	98.31
	S1 (MobileNet-V2)	0.48	738,658	2.82	65.13	98.33

It is important to clarify that the reported inference times in Table 11 reflect the deep learning model's forward pass only. The pre-processing step removes empty patches via intensity thresholding, but adds a negligible computational cost and is therefore not included in the reported latency measurements.

T1 with its DenseNet architecture required the highest training cost while T2 had more parameters. The student achieved near-teacher performance using only a fraction of the computational resources making it suitable for portable and embedded medical systems. GFLOPs were also measured to capture the number of floating-point operations needed for a single forward pass. This metric provides a more accurate measure of computational cost than parameter count alone because it considers both network depth and feature-map dimensions. As expected T1 and T2 had much higher GFLOP values across all resolutions due to dense connections and deeper convolutional blocks. In contrast the MobileNet-V2 student maintained very low GFLOP requirements ranging from 0.12 to 0.48 GFLOPs consistent with its lightweight inverted residual design. The combination of low GFLOPs small model size and competitive accuracy shows that the student model is well suited for environments where computational efficiency and fast inference are essential.

### 5.11 Comparison with previous studies

Table 12 compares the proposed framework with recent state-of-the-art approaches on the GasHisSDB dataset. Conventional CNN-based methods such as VGG16, ResNet50, and LGFFN-GHI generally achieved accuracies in the range of 95–97%, while lightweight backbones like ShuffleNetV2 (MCLNet) demonstrated slightly better performance (up to 97.95%). More recent ensemble or hybrid approaches, including InceptionV3-based models and CNN–Transformer ensembles, pushed performance close to 98.8% but at the cost of significantly higher computational demands. Within this study, the best-performing teacher (ResNet-50) reached an accuracy of **98.31%** on 160 × 160 patches. Remarkably, the distilled MobileNet-V2 (S1) achieved **98.33%** accuracy at the same resolution, slightly surpassing its teacher while maintaining a smaller parameter size (2.82 MB vs. 91.98 MB for ResNet-50). This confirms the effectiveness of the 2T–1S knowledge distillation framework. Compared to prior studies, the proposed S1 model balances high diagnostic accuracy with computational sustainability.

**Table 12:** Comparison of the proposed framework with previous studies on the GasHisSDB dataset.

**References**	**Model details**	**80 × 80 Acc. (%)**	**120 × 120 Acc. (%)**	**160 × 160 Acc. (%)**
Hu et al. (16)	VGG16 / ResNet50	96.12 / 96.09	96.47 / 95.94	95.90 / 96.09
Springenberg et al. (25)	InceptionV3+ResNet50	–	–	98.83 / 98.80
Li and Liu (37)	LGFFN-GHI	–	–	96.81
Fu et al. (38)	MCLNet	96.28	97.95	97.85
Yong et al. (17)	Deep Ensemble	97.72	98.69	99.20
Proposed study	T1 (DenseNet-121)	96.44	**98.75**	98.19
Proposed study	T2 (ResNet-50)	96.63	97.87	**98.31**
Proposed study	S1 (MobileNet-V2)	95.78	97.11	**98.33**

### 5.12 Comparison with other lightweight architectures

To justify the choice of MobileNet-V2 for the student network several contemporary lightweight architectures were evaluated including EfficientNet-Lite0 and MobileNet-V3 (Small). Table 13 shows that MobileNet-V2 achieved the highest accuracy of 98.33% at the 160 × 160 resolution. EfficientNet-Lite0 reached an accuracy of 96.85% while offering no clear accuracy benefit compared to MobileNet-V2.

**Table 13:** Performance comparison of lightweight student architectures on GasHisSDB (160 × 160).

**Student architecture**	**Accuracy (%)**	**Training stability**	**Reason for exclusion / selection**
MobileNet-V3 (Small)	97.10%	Low (Gradient Fluctuations)	Lower accuracy; unstable convergence
EfficientNet-Lite	96.85%	High	Higher latency with no accuracy advantage
**MobileNet-V2 (selected)**	**98.33%**	High	Best accuracy; strong edge-device support

To further evaluate the student model benchmarking of MobileNet-V3 (Small) is also performed. MobileNet-V3 consistently underperformed MobileNet-V2, achieving a maximum accuracy of 97.10% and exhibiting noticeable instability during training. MobileNet V3 struggles here because Squeeze-and-Excitation (SE) blocks add dynamic input dependent channel recalibration that disrupts the stable training flow. In contrast, the teacher models employed in this study (ResNet-50 and DenseNet-121) rely on static convolutional representations without explicit attention mechanisms. During knowledge distillation this mismatch between the student's dynamic feature modulation and the teachers' static supervisory signals can lead to gradient oscillations and prevents stable convergence. MobileNet V2 represents the most suitable student architecture for the framework. The inverted residual structure aligns with teacher feature representations effectively supporting the choice for the proposed distillation framework in the results.

### 5.13 Cross-Dataset validation on DigestPath

To assess robustness under domain shift the student model was tested on the DigestPath 2019 colonoscopy dataset which differs from GasHisSDB. It is important to note that this dataset consists of colon tissue, distinct from the gastric tissue used in training. Results confirm that histological signs transfer well to biologically similar tissues.The work focuses on feature adaptation rather than acting as a multi center gastric cancer validation. Biological differences exist but shared staining and scanner traits allow for a valid transfer learning assessment in the final analysis. Two evaluation settings were used: Zero-Shot inference where the model was applied directly without retraining and Transfer Learning with fine-tuning for 10 epochs. Table 14 shows that Zero-Shot performance ranged from 60.06% to 61.15% across resolutions reflecting the expected domain gap between gastric and colon tissue.

**Table 14:** Evaluation of the student model on the DigestPath dataset under Zero-Shot and fine-tuned settings.

**Resolution**	**Zero-shot accuracy**	**Fine-tuned accuracy (10 epochs)**	**Improvement**
80 × 80	60.06%	92.25%	+32.19%
120 × 120	60.56%	93.29%	+32.73%
160 × 160	61.15%	94.08%	+32.93%

Despite this gap accuracy improved quickly after limited fine-tuning reaching up to 94.08% at 160 × 160 resolution. This shows that the student had already learned transferable histological structures such as nuclear patterns and glandular boundaries that generalized well with minimal adaptation. These results highlight the benefit of domain-specific transfer learning. While ImageNet pre-training remains standard in medical deep learning initializing from a student model trained on a related histopathology domain provides a more effective and computationally efficient starting point. This domain-aligned initialization allowed the model to adapt to the colonoscopy dataset with shorter training time and improved stability.

### 5.14 Statistical analysis and robustness

To assess the statistical reliability of the proposed framework five independent training runs were performed using different random seeds. Results are reported as the Mean ± Standard Deviation (SD) with the 95% Confidence Interval (CI). Cohen's Kappa (κ) was also computed to evaluate agreement with ground truth beyond chance. Table 15 shows that the student model exhibits highly stable and reproducible behavior. The standard deviation across runs stayed below 0.15% for all metrics and the narrow confidence intervals confirm that performance is not sensitive to initialization. The Cohen's Kappa score of 0.966 ± 0.003 indicates near-perfect agreement with expert annotations.

**Table 15:** Statistical reliability of the Student model (160 × 160) over five independent runs.

**Metric**	**Mean value (±SD)**	**95% confidence interval**
Accuracy	98.33%±0.12%	[98.21, 98.45]
Sensitivity	98.39%±0.15%	[98.24, 98.54]
Specificity	98.27%±0.11%	[98.16, 98.38]
Cohen's Kappa (κ)	0.966 ± 0.003	[0.963, 0.969]

### 5.15 Interpretability with integrated gradients

To ensure the transparency of model predictions, interpretability was assessed using Integrated Gradients (IG) (19). IG attributes the prediction of a deep model to its input features by computing the path integral of the gradients between a baseline image and the input. Unlike gradient-based methods, IG provide more reliable explanations of features influenced the classification outcome.

**Figure 15:**
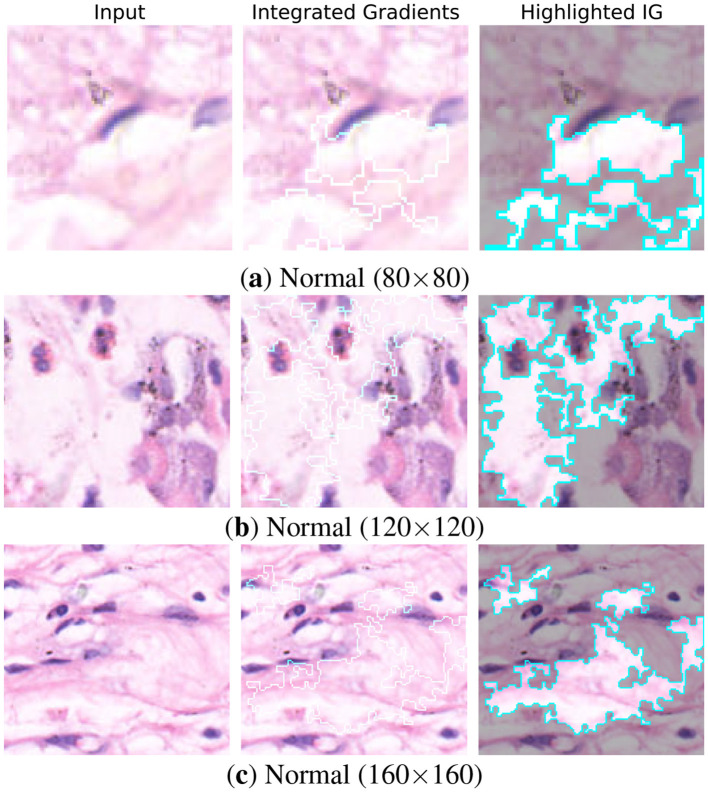
Integrated Gradient visualizations of the Student (MobileNet-V2) for Normal patches across different resolutions: **(a)** Normal (80 × 80), **(b)** Normal (120 × 120), and **(c)** Normal (160 × 160).

**Figure 16:**
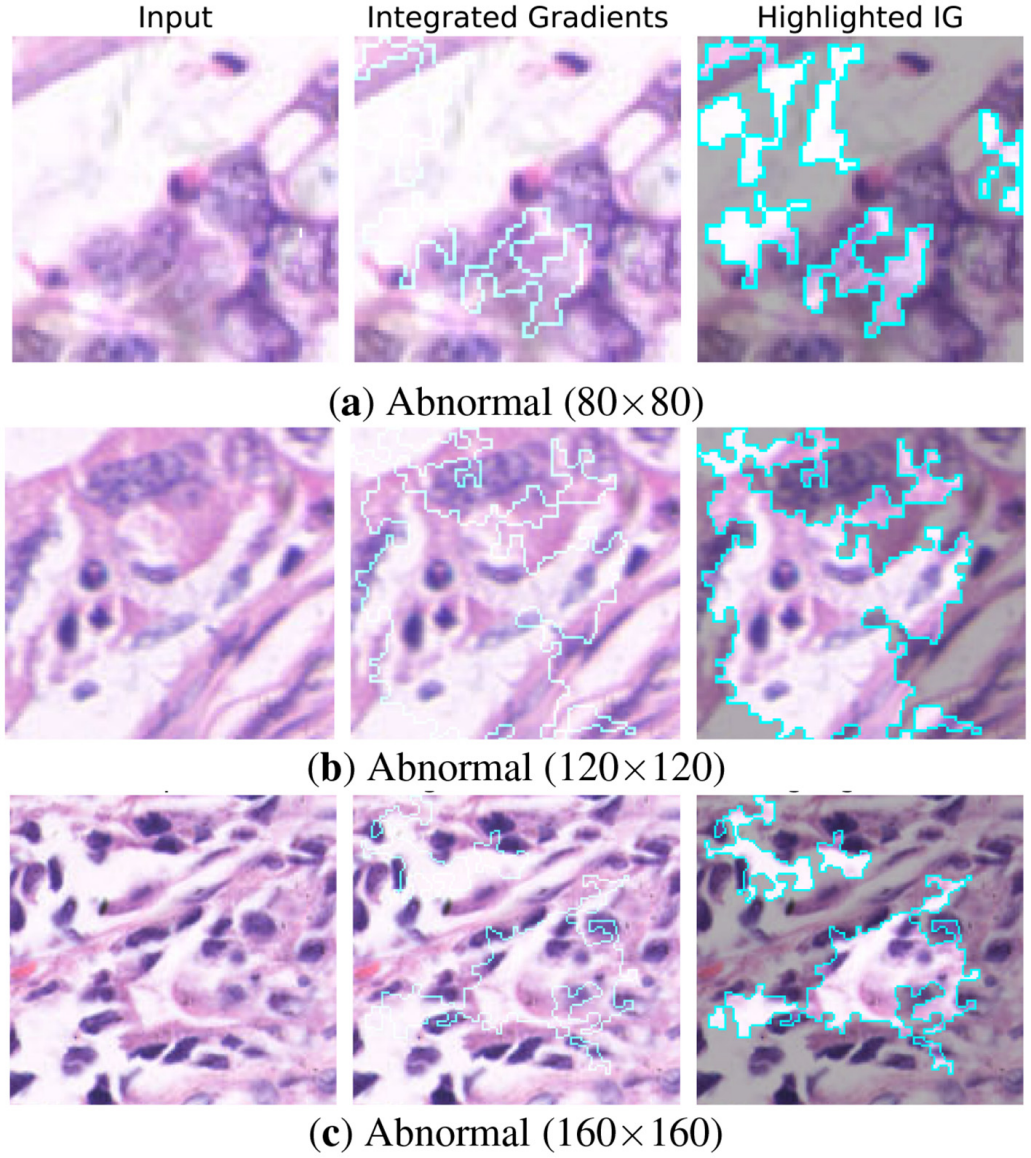
Integrated gradient visualizations of the Student (MobileNet-V2) for Abnormal patches across different resolutions: **(a)** Abnormal (80 × 80), **(b)** Abnormal (120 × 120), and **(c)** Abnormal (160 × 160).

Figures 15, 16 present IG visualizations for normal and abnormal gastric tissue patches across all resolutions. In normal tissue the student model consistently targeted mucosal boundaries and glandular structures. These regions are diagnostically significant because they display preserved cellular organization. The attention in abnormal tissue shifted to nuclei-dense areas and irregular glands. The model also highlighted disrupted morphology which is a hallmark of malignancy. Importantly, the highlighted patterns were consistent across all three resolutions, indicating that the student captured both local cellular details (80 × 80) and broader contextual cues (160 × 160).

The student model closely mirrors the teachers' attributions even with far fewer parameters. This shows that the knowledge distillation framework transfers not only predictive performance but also diagnostic reasoning.Clinically this ensures the model avoids the black box effect. These IG results indicate that the distilled MobileNet-V2 maintains interpretability and more efficient than its teachers.By highlighting relevant details across all resolutions, the model demonstrates its potential as a reliable tool for digital pathology.

**Figure 17:**
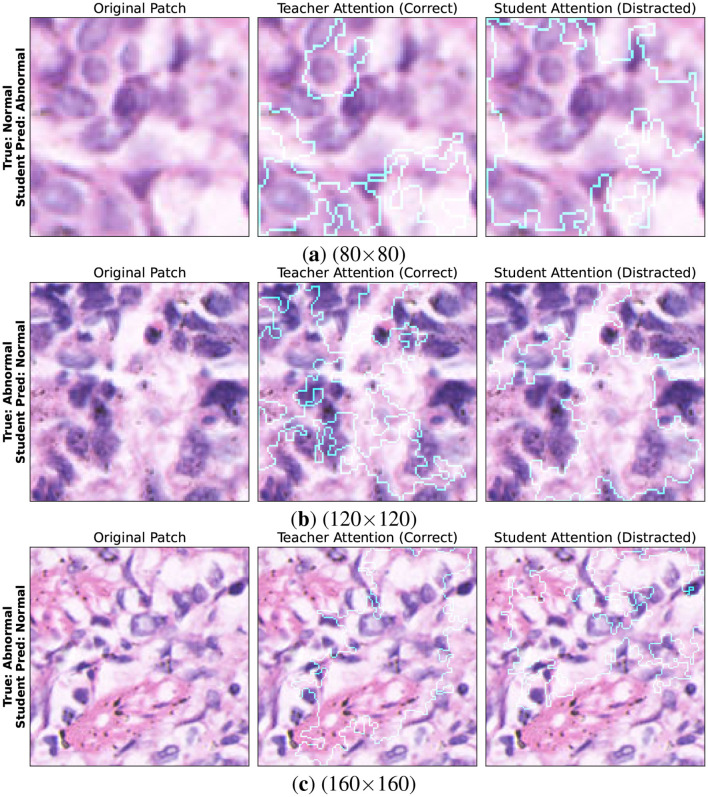
Failure analysis using integrated gradients showing attention mismatch between teachers and student (MobileNet–V2).): **(a)** 80 × 80, **(b)** 120 × 120, and **(c)** 160 × 160.

To further investigate the limitations of the student model, Integrated Gradients are also deployed to analyse hard samples. Hard samples are image patches that were misclassified by the student but correctly predicted by the teacher models. Figure 17 illustrates three representative failure cases at the patch level. The first column presents the original histopathology tissue patches. The second column visualizes the attention maps of the Teachers model (ResNet–50 or DenseNet-121) which correctly highlights relevant regions. The third column shows the attention maps of the Student model (MobileNet–V2) revealing notable deviations in focus. The student fails to detect subtle nuclear and transitional glandular structures in false negative samples. Attention targets benign stromal regions improperly instead. False positive errors occur as hyper-cellular areas mislead the student. Such regions mimic malignant patterns but lack any true pathological significance significance. These observations suggest that although the lightweight student model successfully captures dominant diagnostic features. Reduced representational capacity can result in attention drift under complex boundary conditions, particularly in visually misleading regions.

Since the GasHisSDB dataset does not provide pixel-level annotations. Interpretability was evaluated by measuring the alignment between student and teacher attributions. Structural Similarity Index (SSIM) scores were calculated between the IG of the student and each teacher. Table 16 shows that the student achieved high attribution similarity with both teachers across all evaluated resolutions (SSIM >0.80). These scores suggest the student replicates the teachers' focus. It consistently targets key diagnostic regions such as gland boundaries and nuclear formations.

**Table 16:** Quantitative attribution consistency (SSIM) between student and teacher IG saliency maps.

**Patch Resolution**	**SSIM (Student vs. DenseNet-121)**	**SSIM (Student vs. ResNet-50)**	**Interpretation**
80 × 80	0.82 ± 0.04	0.81 ± 0.05	High spatial consistency
120 × 120	0.85 ± 0.03	0.83 ± 0.04	High spatial consistency
160 × 160	0.88 ± 0.02	0.86 ± 0.03	High spatial consistency

The quantitative comparison indicated an average Structural Similarity Index (SSIM) of 0.88 between the teacher and student attention maps. Qualitative analysis of the high SSIM score confirms the student aligns with the teacher. The model outlines specific borders of malignant glands and nuclear clusters instead of just the general area. High structural fidelity proves the student learned fine grained diagnostic attention patterns from the heavy teacher models avoiding reliance on false background correlations.

### 5.16 Clinical integration and privacy considerations

The proposed framework is designed for telepathology workflows as a pre-screening tool. It identifies high-risk patches with 98.39% sensitivity allowing urgent cases to be prioritized for pathologist review and improving workflow efficiency. The student model is lightweight at 2.82 MB and can perform inference directly on hospital devices. Unlike cloud-based systems that require sending patient data to external servers the framework runs locally on workstations or digital microscopes. This keeps patient slides within the hospital firewall and ensures compliance with privacy regulations.

While the model achieves robust performance with a specificity of 98.27%, this implies a false positive rate of approximately 1.7%. In a pre-screening triage workflow high sensitivity is prioritized to ensure that no potential malignancies are overlooked. These few false positives represent clinical noise. A pathologist rapidly filters the cases during the verification process in the workflow. Moreover, the operating threshold of the model is adjustable, enabling specificity to be further increased when higher diagnostic certainty is required. This flexibility allows the framework to be tailored to different clinical deployment scenarios.

## 6 Conclusion

This study presents a two-teacher one-student (2T–1S) knowledge distillation framework for classifying histopathology images using the GasHisSDB dataset. Knowledge from DenseNet-121 (T1) and ResNet-50 (T2) is transferred to a lightweight MobileNet-V2 student (S1) to achieve competitive accuracy across multiple patch sizes while greatly reducing computational load. For patches of size 80 × 80, 120 × 120 and 160 × 160 the student has fewer than one million parameters and a model size of about 2.8 MB. Inference takes 61 to 65 milliseconds per patch and accuracy reaches 95.78% 97.11% and 98.33% respectively. These results suggest that S1 can match or sometimes surpass its teachers while being far more efficient. Integrated Gradients show that the student consistently focuses on relevant structures such as nuclei clusters and gland boundaries. This interpretability can build trust and support pathologist validation. Compared with T1 and T2 S1 achieves a good balance between accuracy speed and model size. It is well suited for deployment in cloud platforms or hospital laboratories. Future work could explore multi-student ensembles, incorporation of multimodal data such as genomic or clinical features, and optimization for embedded hardware. These directions are likely to extend the practical impact of knowledge distillation in digital pathology and enable more sustainable AI in clinical workflows.

## Data Availability

Publicly available datasets were analyzed in this study. This data can be found here: Gastric Histopathology Sub-database (GasHisSDB) (16). The dataset is openly available at https://gitee.com/neuhwm/GasHisSDB and is also hosted on Figshare (DOI: 10.6084/m9.figshare.15066147). Additionally, a downloader script is available in the GitHub repository at https://github.com/engrkhubabahmad/gashissdb-dataset.
